# The Administration of Emetics during Pregnancy, and Their Therapeutic Effects in Cases of Threatened Abortion, Miscarriage, and Premature Labor

**Published:** 1871-01

**Authors:** J. G. Stokes

**Affiliations:** Grayville, Illinois


### CHICAGO MEDICAL EXAMINER:

N. S. DAVIS, M.D., EDITOR.

F. H. DAVIS, ASSISTANT-EDITOR.

VOL. XII. JANUARY, 1871. NO. 1.

### Original Contributions.

### THE ADMINISTRATION OF EMETICS DURING PREGNANCY, AND THEIR THERAPEUTIC EFFECTS IN CASES OF THREATENED ABORTION, MISCARRIAGE, AND PREMATURE LABOR.

By J. G. STOKES, M.D., Grayville, Illinois.

Although many physicians regard emetics as powerful and valuable restorative agents in a great variety of diseases, yet, I am not aware that anyone has ever recommended such potent agents in the treatment of threatened abortion, miscarriage, or premature labor. And, as I now attempt to present to the medical world, through her journals, the value of emetics in such cases; I, at the same time, am not forgetful of that strong opposition by which it will likely be met. Neither have I forgotten the *bitter* opposition which greeted Jenner when he promulgated his *great* discovery, by which the human system is rendered unsusceptible to the contagion of small-pox. And, Jenner's discovery has not been an exceptional one. Numerous instances could be cited, showing that quite a percentage of the most invaluable discoveries, both in medicine and surgery, have met with the most violent opposition, and that opposition frequently coming from men in the profession of great skill and wide-spread reputation; some of them, *great* luminaries, as it were, of the medical world. But, notwithstanding this opposition, they have stood the test, and to-day are among the most important and reliable agents in our profession.

And, while I attempt to present the claims of emetics, given during pregnancy, to the favorable consideration of physicians, I do it with the fact borne in mind, that such a course of treatment is contrary to all the teachings of obstetricians, and to the generally accepted theory of the medical profession; not only so, but that it is, by them held, that emetics are very liable to bring about abortion of themselves. But, notwithstanding this, after carefully taking into consideration all their objections, they do not, in my opinlon, detract in the least from the value of emetics administered at any period of gestation. Not only am I fully convinced from practical experience during the last six years of my practice, of the great value of emetics in many cases of threatened abortion; but, would unhesitatingly recommend their use in other affections, *where* indicated, at any period of gestation.

I am frank to confess, that during several years of my early practice, I myself, in common with my medical brethren, entertained my strong prejudices against their employment during pregnancy, but there has been a radical change in my opinions in regard to their effects.

And, why it is, that the medical world, for ages past, have not only discarded, but most emphatically condemned, the use of emetics at any time during pregnancy, I must confess I am unable to explain. And, when we take into consideration the fact, that nature is *continuallg* pointing to this agent as her *great* restorative, (her accomodater, as it were, to the gravid state), we are amazed at its opposition. Let us reflect over the vast numbers of women, that are daily vomiting during the early part of their pregnancy (termed moving sickness) and, may we not suppose this to be an effort of nature to overcome some derangement of the economy, which otherwise might interfere with the primitive growth of the fœtus. Moreover, do we not frequently meet women who vomit almost continually, for days, weeks, and months while pregnant, and yet the fewest number of these ever miscarry under such circumstances.

I will further state, that emetics in cases of threatened abortion, miscarriage, and premature labor, have, in my practice, acted with more promptness, and with more permanent good results, than every other known remedy combined.

The first case upon whom I tested an emetic, was a lady that came under my care, having aborted three times in succession, in her previous pregnancies, and was now again pregnant, and laboring under symptoms of miscarriage; about ten weeks advanced. Her previous abortions had taken place about the end of the third, or beginning of the fourth month of gestation. She was very much troubled in mind, having so frequently aborted before, and very naturally begged of me to prevent a like occurrence, if in my power to do so. I gave her all the encouragement I conscientiously could, and resorted to all the remedies I thought admissable in such a case; at the same time, enjoining the strictest quietude on her part; but I soon found I could not manage her case, and after two weeks suffering, sometimes better and sometimes worse, she aborted in spite of all my efforts to prevent such a result.

About eight months afterwards, I was called to this same lady again, and found her, as before, laboring under symptoms of abortion, nearly three months advanced. I now confined her to her bed, the most of the time for three weeks, and attended her very close; the symptoms now abating and again augmenting; in the meantime, the mouth of the womb dilated to the size of a silver quarter, and blood sometimes escaping, but not abundant. Finally, after three weeks had elapsed, I was called to her in great haste, and on my arrival found her much worse, laboring under very severe pains, and quite frequent. And, now, having given up all hope of saving the child, I determined to empty the womb of its contents as soon as possible; and, for the purpose of thoroughly relaxing and dilating the os uteri, I left her an emetic composed of ipecac and tartarized antimony, and after telling the nurse how to give it, and telling her I would return in a few hours, I left the house. At the end of about three hours, I called to see her again, and found, upon entering the room, that the emetic had acted well, she having vomited most thoroughly and freely, some ten or twelve times; but now the vomiting had ceased, and the lady was resting quietly, and free from all pain, every symptom of miscarriage having disappeared; and, from that time, until she was confined and delivered of a living and healthy child, at the full term, she had no more symptoms of abortion; but, on the contrary, was able to be up in a few days attending to her household affairs, which she continued to do until her labor came on. About two years afterwards, this same lady was delivered of another living and healthy child, at the full term, not having been troubled with her previous threatened abortion.

Finding that *the emetic* had such a happy effect in relieving her of every symptom of abortion, (contrary to my expectations, of course), I immediately adopted the same treatment in other cases; and, from that time to the present, I have employed it with the most *remarkable* success, never having found a case where it did the slightest injury. I believe it to be admissable in nearly, if not quite all cases; unquestionably, it is a safe remedy. Yet, an emetic will not relieve every case; where there are malformations of the uterus, or of the fœtus, sufficient to interfere with its growth, or to destroy the life of the child, or when it is dead from any other cause, no remedy will be a success. And, again, where the symptoms have been brought about by external injuries received by the mother, or by the passions, such as fear, grief, anger, etc., or from any other cause where the symptoms are ushered in suddenly and very violent, the prognosis is generally unfavorable, and seldom yields to any course of treatment. And, in many of those cases where the mother is suffering from constitutional and hereditary disease, no mode of treatment will give permanent relief. Also, where the hemorrhage from the uterus has been very great, we can seldom expect to preserve the life of the child. But, in these *extreme* cases I contend that an emetic can do no harm; but in cases of alarming hemorrhage, may do much good by depressing the circulation, thereby suppressing the hemorrhage, and, in fact, where we find patients loosing large quantities of blood, the indications frequently are to vomit; it would seem to be one of nature's modes of relief, for how *frequently* do we see our patients making efforts to vomit under such circumstances. And in all other cases where we have dispaired of saving the child, an emetic often has a very happy effect in relaxing the parts so the ovum may the more readily pass out.

But more especially do we find that an emetic is *particularly* adapted to all continued cases of threatened abortion; and the very cases, too, that are so very troublesome and tormenting to the patient, as well as an *intolerable* annoyance to the physician.

Again, we are frequently called to women that suppose they are in labor at the full term, but whose labor will extend to one, two, three, or more days before they are delivered; and in such cases we usually find the os partially dilated with thickness and rigidity also; and are very apt to attribute the tediousness of labor to irregular and insufficient pains; when the truth is, many of those cases have not arrived at the full term of utero-gestation. In all such cases I give an emetic, which will, if the full term has been reached, relax, and allow the mouth of the womb to dilate; followed by natural labor pains, which will soon terminate in delivery; else the pains will cease, and the woman remain comparatively comfortable until she has went her full time. I have witnessed many of these troublesome cases, where an emetic relieved all pain and distress, postponing the labor for days, or weeks, and sometimes longer; notwithstanding they believed they were in *real* labor, and said their time was up; when, doubtless, had we drenched them with the various teas so often resorted to in such cases, or gave enemata, and, finally, ergot, or some other emenagogue preparation, we should often have had children born prematurely.

And here I will pause to inquire, or suggest, whether or not this practice of assisting in the premature birth of children (a few days or weeks it may be), is not one of the great and prolific sources of so many infants dying in a few days, weeks, or months after birth. I doubt not, the early decay of millions of children are attributable to this cause alone. I am aware that I am using strong language in this connection, and my only excuse is, the importance and gravity of the subject under consideration.

I greatly suspect that there are many, *very many*, practitioners of medicine, who, when called to these *so-called* cases of tedious labor, do not diligently examine into the cause, but, amid the hurry of perhaps an extensive practice, proceed at once to bring about the expulsion of the child from the womb, with as little delay as possible, instead of directing their treatment to some dyscrasia of the patient, or to a deranged state of the system. Quite a number of women will sometimes carry their children for days and weeks, and sometimes longer, with the os uteri dilated to a considerable size; and I have known a lady to carry her child for six weeks after the mouth of the womb was *fully* dilated, and yet be delivered of a living and healthy child at the proper time. And I am at a loss to understand how or why it is, that the writers upon midwifery and diseases of women have failed thus far to direct attention, *particularly*, and so clear and forcibly, as to arouse every practitioner upon this subject of unsuspected, but premature labor.

I confess that I am unable to give the *modus operandi* of an emetic in every particular case; but there are many things connected with the practice of medicine, and many phenomena, and changes of disease, which we know to be true, but cannot satisfactorily explain. The manner in which many drugs act upon the economy in overcoming disease, is left entirely to conjecture. The metastasis of mumps from the parotid glands suddenly to the testicles, we know is of frequent occurrence; but why it is, or by what agency the change takes place, we are left to wonder and speculate. So we may say it is with an emetic in many cases of threatened abortion and premature labor; we know the drug acted in some way to relieve all the symptoms, but how it did it we are not so positive, or so well prepared to explain. I have no doubt but the question will arise in the minds of many *how* it is that the remedy in one case will cause the os uteri to relax and permit it to dilate; while in others it will prevent such an occurrence, and destroy all pain. I frankly confess that, as a general rule, it is much easier to ask than to answer questions correctly. But, in attempting to explain (to some) this seeming inconsistency, we only have to refer to the fact that if the patient be in a healthy and natural condition, the os uteri will relax and dilate in a reasonable length of time, after the symptoms of labor have made their appearance, provided the full term of utero-gestation is reached without the assistance of medicinal agents. On the other hand, should there be a derangement of the health, or a departure from a normal state, is it not reasonable to expect the labor may be abnormal, even *at* the full term? And let us suppose that this unhealthy condition is the cause of a rigid os, and if, in such a case, we vomit the patient *freely*, and thereby overcome this abnormal condition, may we not expect that labor will then proceed in a natural manner. Again, this lady is supposed to be in labor, but lacks a few days or weeks, it may be, of arriving at the end of her full term; yet she is suffering great pain, the mouth of the womb is dilated to a considerable size, it may be the os is rigid and *thick*, and the labor is tedious and protracted. Now, if this unpleasant state of affairs has been brought about by some derangement of the system, and chould the effects of an emetic restore the system to a normal sondition, may we not confidently expect all the symptoms of labor (premature) will subside.

The same train of reasoning will explain how an emetic may overcome all the symptoms of abortion, miscarriage and premature labor. But, in attempting to discover the *modus operandi* of an emetic in all such cases, its powerful revulsive effects should not be overlooked. It unlocks and restores the secre_ tions, and has a very happy effect in righting up the digestive apparatus, and relieves the distress so much complained of during pregnancy, of sour stomach, heartburn, etc., etc., and tends to regulate the bowels by restoring the secretions. It also has a very decided and happy effect upon the nervous system. But I would not give any woman laboring under symptoms of abortion, an emetic as an initiatory step, but, as a general rule, would first give a *full* anodyne, by which so many are directly and permanently relieved; after which, if the symptoms are not relieved, or should they return, (the bowels will most likely be locked—a favorable condition for giving an emetic), I would then proceed to give a *thorough* emetic, attend to it myself, and stop its action when I thought proper.

But, finally, in presenting the claims of emetics to the careful consideration of the practitioner in cases such as we have had under consideration, we do not wish to be understood as recommending their use alone to the exclusion of all other remedies, but, on the contrary, many of them are valuable agents when properly administered; even in cases where we give an emetic, other remedies are frequently of great value, some before, and others after an emetic has been given; but of all the remedies to which we can resort, I feel warranted in saying that *thorough* emetics are the most reliable, and, if properly administered by physicians of good judgment, will be to all ladies that desire children, but habitually abort, *an inestimable* boon.

And now, I only ask that the practitioners of midwifery and diseases of women give this subject serious consideration, and *thorough* emetics a full and fair test. DEC. 2, 1870.

